# Supplementary material for: Na0.76V6O15/Activated Carbon Hybrid Cathode for High-Performance Lithium-Ion Capacitors
Source: Materials (Basel). 2020 Dec 30;14(1):122. doi: 10.3390/ma14010122 (PMC7794966; doi:10.3390/ma14010122)
Supplement: Supplementary file 1 [file materials-14-00122-s001.pdf]

Supplemental information

$Na_{0.76}V_6O_{15}$/Activated Carbon Hybrid Cathode for High-Performance Lithium-Ion Capacitors

Renwei Lu [1], Xiaolong Ren [1], Chong Wang [1], Changzhen Zhan [1], Ding Nan [2,*], Ruitao Lv [1,3], Wanci Shen [1], Feiyu Kang [1,3] and Zheng-Hong Huang [1,3,*]

1 State Key Laboratory of New Ceramics and Fine Processing, School of Materials Science and Engineering, Tsinghua University, Beijing 100084, China; thulrw@163.com (R.L.); 18801291089@163.com (X.R.); wang-c18@mails.tsinghua.edu.cn (C.W.); zcz@gmomi.com (C.Z.); lvruitao@tsinghua.edu.cn (R.L.); shenwc@mail.tsinghua.edu.cn (W.S.); fykang@sz.tsinghua.edu.cn (F.K.)

2 China College of Chemistry and Chemical Engineering, Inner Mongolia University, Hohhot 010021, China

3 Key Laboratory of Advanced Materials (MOE), School of Materials Science and Engineering, Tsinghua University, Beijing 100084, China

* Correspondence: zhhuang@tsinghua.edu.cn (Z.-H.H.), nan1980732@163.com (D.N.)

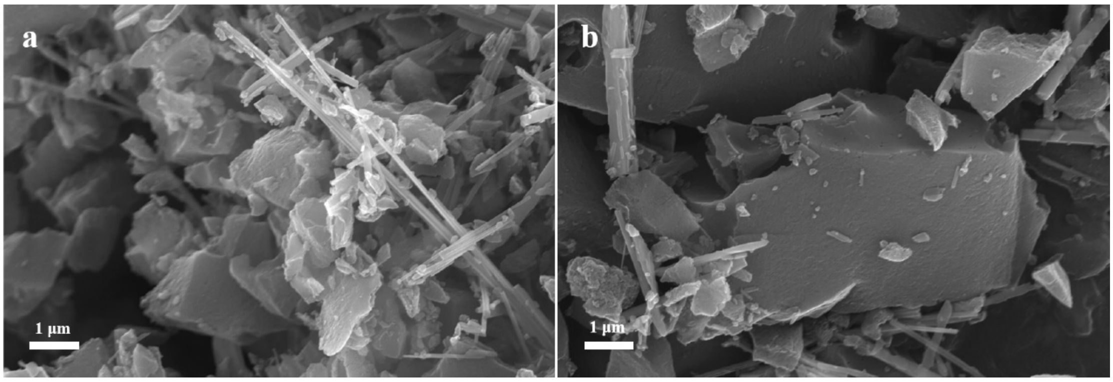

Figure S1. SEM images of the (**a**) 20-NaVO/C and (**b**) 30-NaVO/C hybrids.

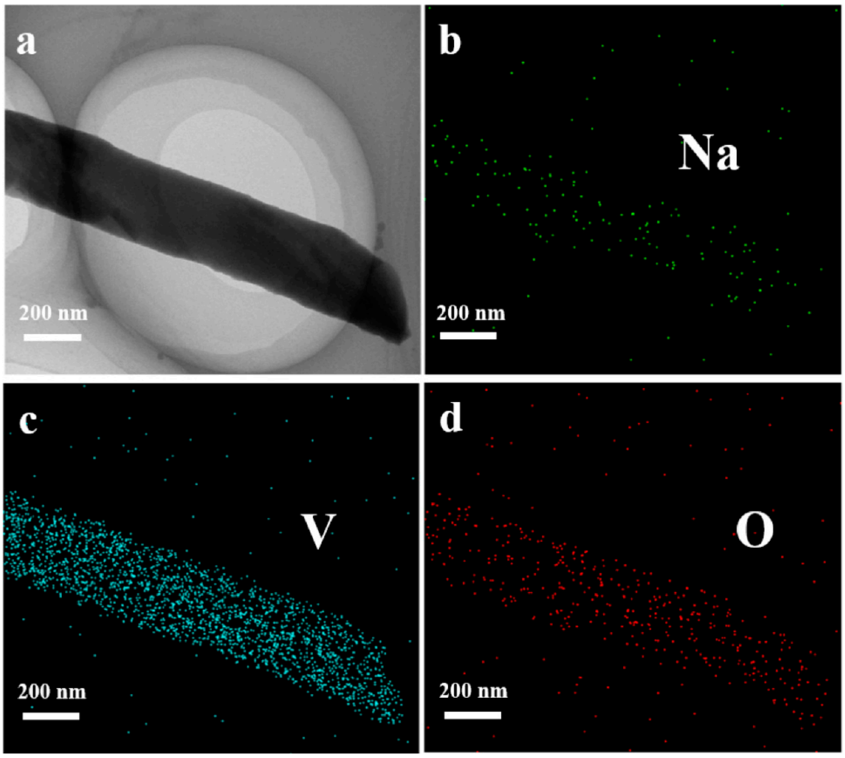

Figure S2. Corresponding elemental distribution mapping of NaVO nanobelts. TEM image of (**a**) NaVO; (**b**) Na; (**c**) V; (**d**) O.

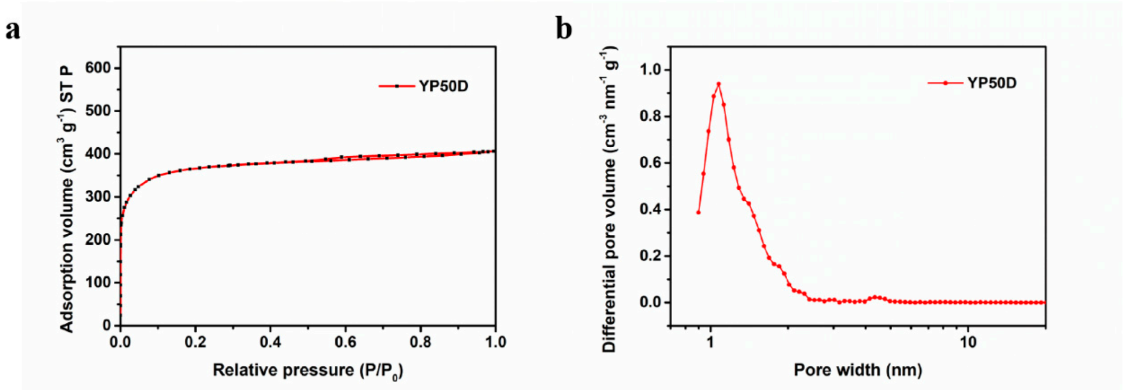

Figure S3. (**a**) N2 adsorption/desorption isotherms of YP50D; (**b**) pore-size distribution curves of YP50D calculated based on density functional theory method.

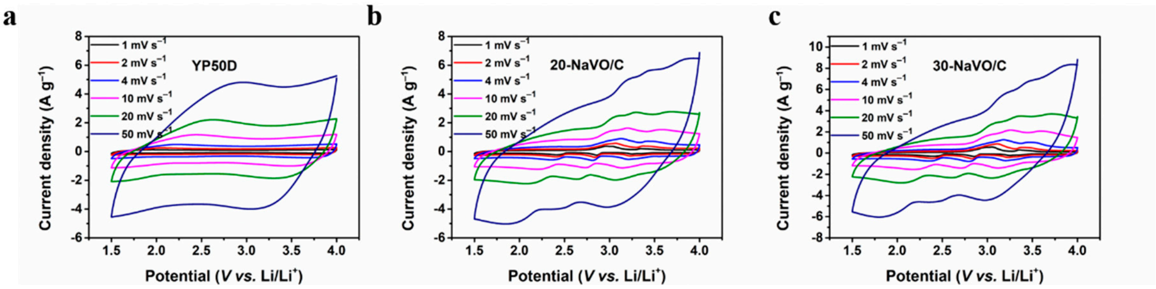

Figure S4. CV curves at different scan rates from 1.5 V to 4 V. (**a**) YP50D; (**b**) the 20-NaVO/C hybrid; (**c**) the 30-NaVO/C hybrid.

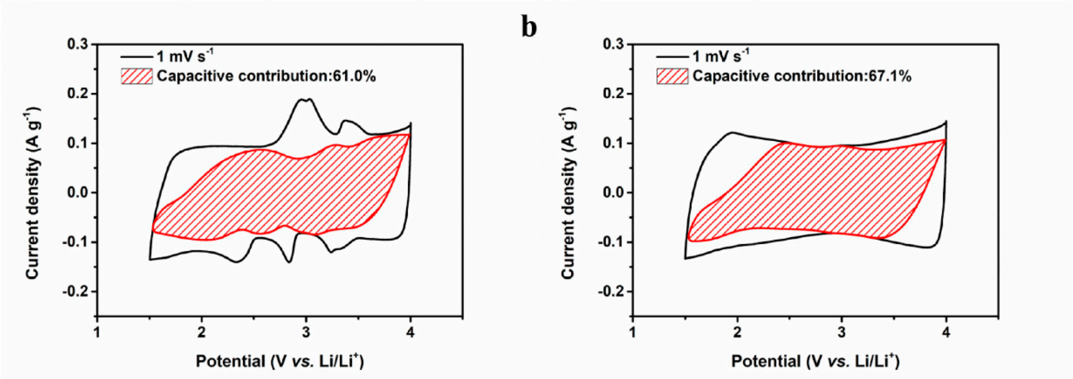

Figure S5. Capacitive contribution area in the CV curve at the scan rate of 1 mV s^{-1}. (**a**) 10-NaVO/C; (**b**) YP50D.

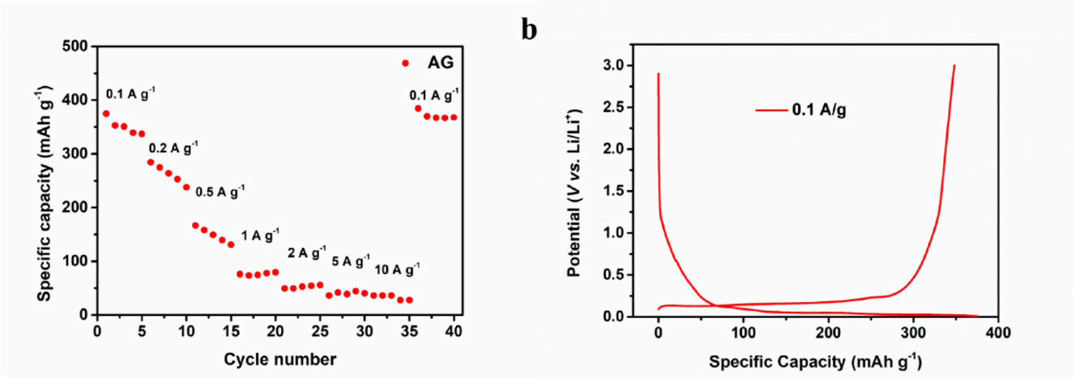

Figure S6. Electrochemical performance of artificial graphite (0.01–3 V) (**a**) rate performance at different current densities from 0.1 to 10 A g^{-1}; (**b**) GCD curve for the first cycle at 0.1 A g^{-1}.

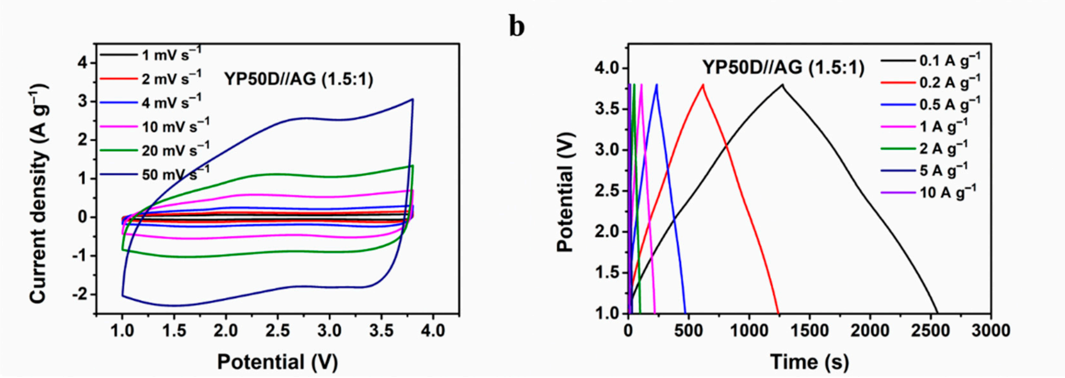

Figure S7. (**a**) CV curves of YP50D//AG (1.5:1) at different scan rates; (**b**) GCD profiles of YP50D//AG (1.5:1) at different current densities.

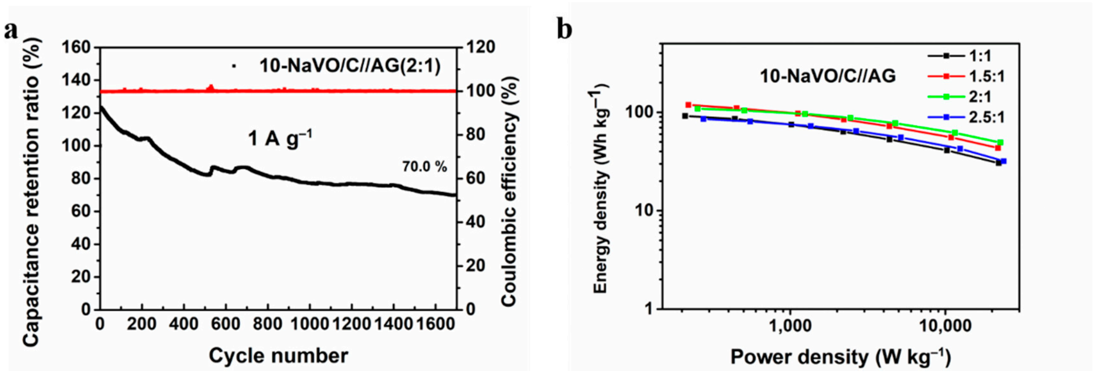

Figure S8. (**a**) Cycling performance of 10-NaVO/C//AG (2:1) at 1 A g^{-1}; (**b**) Ragone plots of 10-NaVO/C//AG with different cathode-to-anode mass ratios.
